# Reply to Jaeschke and Ramachandran, “Putative Effects of an Orally Administered Anti-TLR4 Antibody on Gut Microbiota and Acetaminophen Hepatotoxicity in Mice”

**DOI:** 10.1128/spectrum.02377-22

**Published:** 2022-12-13

**Authors:** Xuewei Sun, Qian Cui, Juan Ni, Xiaoguang Liu, Jin Zhu, Tingting Zhou, HuaYing Huang, Ke OuYang, Yulong Wu, Zhan Yang

**Affiliations:** a Centre for Diseases Prevention and Control of Eastern Theater, Nanjing, China; b Binzhou Medical University, Yantai, China; c Air Force Hospital of Eastern Theater, Nanjing, China; d The First Affiliated Hospital of Nanjing Medical University (Jiangsu Province Hospital), Nanjing, China; e Department of Respiratory and Critical Care Medicine, Jinling Hospital, The First School of Clinical Medicine, Southern Medical University, Nanjing, China; University of Nebraska—Lincoln

## REPLY

We sincerely appreciate the letter from you asking for more information about our work. Dr. Jaeschke and Dr. Ramachandran asked some questions which are useful for us to address. We will answer the questions as follows.

In the first question, Dr. Jaeschke and Dr. Ramachandran pointed out that orally gavaged antibody was unlikely to be effective. We tried two methods for applying antibody, by oral gavage and intraperitoneal injection in our preexperiment. However, the results of the oral gavage were not effective. So, we chose the intraperitoneal injection as the administration method for our paper. However, when we wrote the article, we made a mistake in that we wrote in the paper that antibody was administered by using the oral gavage method. We decided to retract the paper to address the mistake. The retraction of the article can be found at https://doi.org/10.1128/spectrum.03116-22.

In the second question, Dr. Jaeschke and Dr. Ramachandran said that liver injury will rapidly happen when an excess of APAP is used, and it will be within a strict window. However, in this process, according to our experimental results, which compared with liver cells and changes of the gut microbiota, its metabolites and the intestinal barrier, APAP injury may not rapidly happen, and the related changes of the gut may last for a period of time.

In the third question, as Dr. Jaeschke and Dr. Ramachandran noted that “by using fecal microbiota transplantation for 3 days,” it means that in the process of fecal microbiota transplantation, the transplantation of fecal microbiota of the donor to the receptor needs 3 days, and the aim is to ensure that the gut microbiota of the receptor and the donor stay the same. For short-chain fatty acids, the well-studied metabolites of gut microbiota, in many studies, the intervention period is generally long; for example, in the study of Zhu et al. (1), the intervention period is 14 days. Our pre-experiment result showed that the intervention period for 7 days can also get the expected result. So, we fed short-chain fatty acids for 7 days in this article. As described above, the application method of antibody is according to the research foundation of our laboratory. While we studied the antibody according to the method of one application, this question also reminds that more research is needed about the intervention time point and intervention period. This can become one of the directions in our following research.
